# Supplementary material for: Phylogeographic analysis of Begomovirus coat and replication-associated proteins
Source: J Gen Virol. 2024 Oct 24;105(10):002037. doi: 10.1099/jgv.0.002037 (PMC11500754; doi:10.1099/jgv.0.002037)
Supplement: Uncited Supplementary Material 1. [file jgv-105-02037-s001.pdf]

Table S1. Begomovirus species exemplars listed in the VMR that were not used in this study.

VMR exemplars not used in this study (RefSeq accession number)	Reason
Bean golden yellow mosaic virus (NC_038791)	Truncated Rep
Chino del tomate Amazonas virus (NC_038443)	Significantly divergent Rep
Chilli leaf curl Bhavanisagar virus (NC_055130)	Truncated Rep
Cleome golden mosaic virus (NC_015397)	Significantly divergent and truncated Rep
Corchorus yellow vein mosaic virus (NC_020473)	Truncated Rep
Polygala garcinii virus (NC_037068)	Significantly divergent Rep
Sunn hemp leaf distortion virus (NC_013019)	Both CP and Rep listed as nonfunctional in GenBank (sequences not annotated as a result)
Sidastrum golden leaf spot virus (NC_038462)	Significantly divergent CP
Sida golden yellow spot virus (NC_038992)	Significantly divergent CP
Tomato golden leaf distortion virus (NC_043122)	Significantly divergent Rep
Tomato leaf curl Joydebpur virus (NC_074895)	Truncated CP and Rep
Tomato leaf curl Moheli virus (NC_038897)	Truncated Rep
West African Asystasia virus 3	Does not have a RefSeq accession

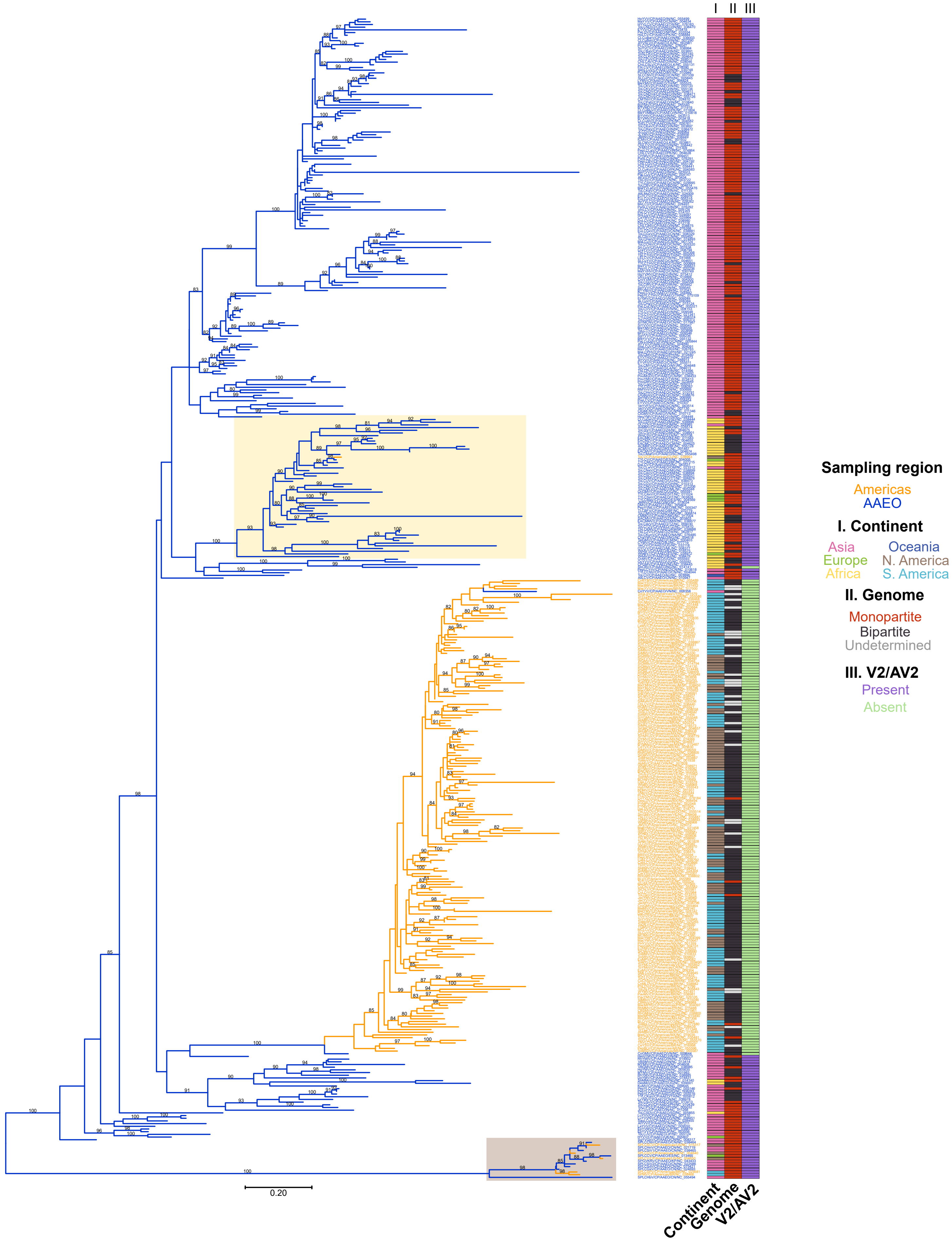

Figure S1. Midpoint-rooted maximum likelihood phylogenetic tree of complete CP amino acid sequences of 432 begomovirus RefSeq species exemplars. The maximum likelihood phylogenetic tree was constructed using IQ-Tree v2.07 with automatic selection of the best-fit substitution model (JTT+I+G4). Tree inference was performed with 3000 ultrafast bootstrap (UFBoot) replicates and a stopping rule of 500 iterations between unsuccessful improvements to the local optimum. UFboot branch support values ≥ 80% are shown mid-branch. The scale bar represents the number of substitutions per site. Branches are colored based on the region where the exemplar was sampled - Americas exemplars in orange and AAEO exemplars in blue. Sweepoviruses (light brown box) and the African clade (yellow-orange box) are highlighted. Exemplar labels follow the following format: "virus abbreviation/Rep/[Americas/AAEO]/two-letter country code/RefSeq accession number".

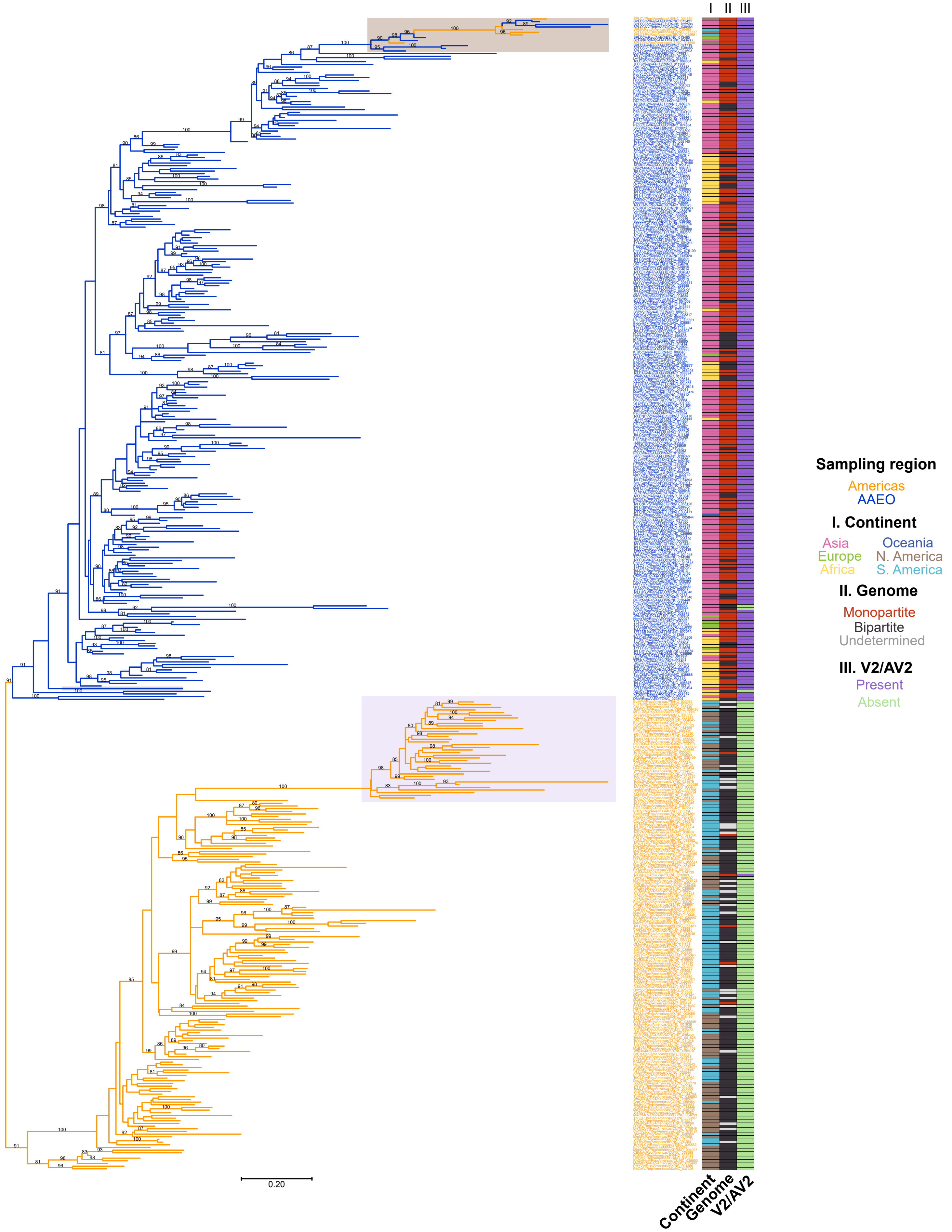

Figure S2. Midpoint-rooted maximum likelihood phylogenetic tree of trimmed Rep amino acid sequences of 432 begomovirus RefSeq species exemplars. The maximum likelihood phylogenetic tree was constructed using IQ-Tree v2.07 with automatic selection of the best-fit substitution model (LG+I+G4). Tree inference was performed with 3000 ultrafast bootstrap (UFBoot) replicates and a stopping rule of 500 iterations between unsuccessful improvements to the local optimum. UFboot branch support values ≥ 80% are shown mid-branch. The scale bar represents the number of substitutions per site. Branches are colored based on the region where the exemplar was sampled – Americas exemplars in orange and AAEO exemplars in blue. Sweepoviruses (light brown boxes) and Rep S-Lin clade (light purple box) are highlighted. Exemplar labels follow the following format: "virus abbreviation/Rep/[Americas/AAEO]/two-letter country code/RefSeq accession number".
